# Discrete Motor Coordinates for Vowel Production

**DOI:** 10.1371/journal.pone.0080373

**Published:** 2013-11-14

**Authors:** María Florencia Assaneo, Marcos A. Trevisan, Gabriel B. Mindlin

**Affiliations:** 1 Dynamical Systems Lab, Physics Department, University of Buenos Aires, Buenos Aires, Argentina; 2 Integrative Neurocience Lab, Physics Department, University of Buenos Aires, Buenos Aires, Argentina; Northwestern University, United States of America

## Abstract

Current models of human vocal production that capture peripheral dynamics in speech require large dimensional measurements of the neural activity, which are mapped into equally complex motor gestures. In this work we present a motor description for vowels as points in a discrete low-dimensional space. We monitor the dynamics of 3 points at the oral cavity using Hall-effect transducers and magnets, describing the resulting signals during normal utterances in terms of active/inactive patterns that allow a robust vowel classification in an abstract binary space. We use simple matrix algebra to link this representation to the anatomy of the vocal tract and to recent reports of highly tuned neuronal activations for vowel production, suggesting a plausible global strategy for vowel codification and motor production.

## Introduction

Phonetics comprises different strategies for transforming the sounds of speech to different spaces. One of them is the mapping of vowels to the first two vocal tract resonant frequencies *F*_1_ and *F*_2_, where the different vowels cluster into coarse regions of the space (*F*_1_, *F*_2_) [1]. Another well-known map classifies the vowels in terms of the height and backness of the tongue, which is the basis of the International Phonetic Alphabet, IPA [2]. The basic idea underlying these canonical approaches is that vowels can be associated with functional perturbations of the vocal tract, a series of cavities extending from the glottal exit to the mouth, whose shape can be actively modified by the action of major articulators like the tongue, jaw and lips.

Vowels are produced by the combined action of the vocal tract and the vocal folds (flexible tissue membranes located at the glottis) as follows: the vocal folds are set into oscillatory motion by the transfer of energy from the airflow expelled from the lungs, and the perturbed airflow produced by this oscillation is injected into the vocal tract, that acts as a waveguide for the sound.

Although the utterance of vowels results from the combined action of the vocal tract and vocal folds, both blocks act rather independently during normal speech, because the folds are not appreciably affected by the re-injection of sound from the tract, which is known as source-filter theory [1]. The main consequence of this uncoupling is that the spectrum of a vowel is characterized by acoustic features exclusively associated with the vocal folds, as the fundamental frequency *f*_0_ (pitch), and others associated with the vocal tract, as the resonant frequencies *F*_i_ (formants). From a phonetic point of view, the vocal folds represent a passive vehicle of sound, while the vocal tract is the key anatomical element to which phonetic information can be tracked back to. The challenge and success of classical phonetics has been the reduction of the problem to low-dimensional spaces, like the canonical descriptions of vowels using a few vocal tract resonances or positions of the articulators. However, these descriptions still assume the continuous nature of the morphological changes of the vocal tract.

In this work we investigate an alternative to this standard view, describing the Spanish vowels in a discrete binary space from experimental vocal tract data taken during the utterance of vowels.

**Figure 1:**
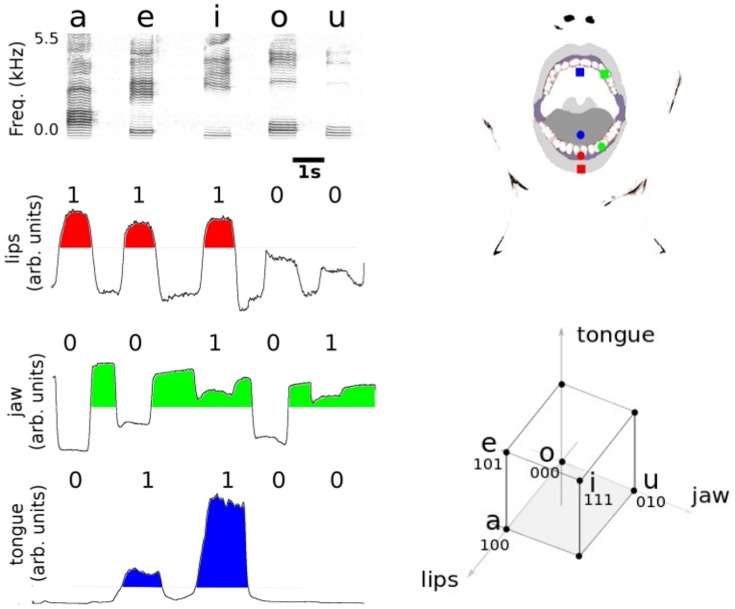
From transducer signals to binary vowel space. Upper right panel: sketch of Hall Effect transducers and magnets in the oral cavity. Transducers are marked with squares and magnets with circles. *Lips* (red): the transducer was attached to the center of the lower lip and the magnet was glued to the dental plastic replica, in between the central incisors. *Jaw* (green): magnet and transducer were glued to the dental replicas, in the space between the canine and the first premolar of the upper and lower teeth respectively. *Tongue* (blue): a cylindrical magnet was attached at a distance of 1.5 cm from the tip of the tongue. The corresponding Hall Effect transducer was glued to the dental plastic replica, at the hard palate, 1 cm right over the superior teeth (sagittal plane). Transducer wire was glued to the plastic replica and routed away to allow free mouth movements. Left, downwards: a spectrogram of the set of 5 vowels as pronounced by one of the subjects during a recording session (and frequency values for the first 2 formants) and the corresponding transducer signals for the lips, jaw and tongue. A binary code for each vowel can be defined by labeling the signal of each articulator as active (1) or inactive (0) as it reaches or not a predefined threshold (areas in color correspond to active motor coordinates). Lower right panel: resulting vowel-cube in the binary space. The edges of the cube represent an abstract space of size 8, were we explicitly locate the 5 vowels used in this work.

We monitor vocal tract dynamics during controlled utterances of isolated vowels using pairs of Hall Effect transducers and magnets mounted in the upper vocal tract. Hall Effect transducers are cheap, light and small detectors that convert changes in the magnetic field to voltage differences, responding to relative distances and orientations of the magnet-transducer pair [3]. We acquired data from the different movements of the articulators by mounting one pair for monitoring the jaw (upper and lower dentures), another one for the dynamics of the tongue (relative to the hard palate), and a third one for the lips (relative to the teeth), as sketched in the upper right panel of Figure 1 (see Methods).

## Results

In the left panels of Figure 1 we show examples of the recorded sound and motor data for one of the participants (audio and transducer files are available at Supplementary Information, files S1 to S4). Each transducer signal either remains at a baseline or responds with a voltage change during the utterance of the different vowels, which leads to a simplified binary description where each transducer acts as a switch between 2 dynamical states. Inactive states (0) become active (1) when signals cross a threshold, like the ones shown in red, green and blue in the left panels of figure 1. In this representation, the vowels read:/a/ = (1,0,0);/e/ = (1,0,1);/i/ = (1,1,1);/o/ = (0,0,0) and/u/ = (0,1,0) for lips, jaw and tongue transducers respectively. In this discrete motor space, an abstract vowel-cube appears whose edges correspond to different Spanish vowels (right panel of Figure 1).

**Figure 2:**
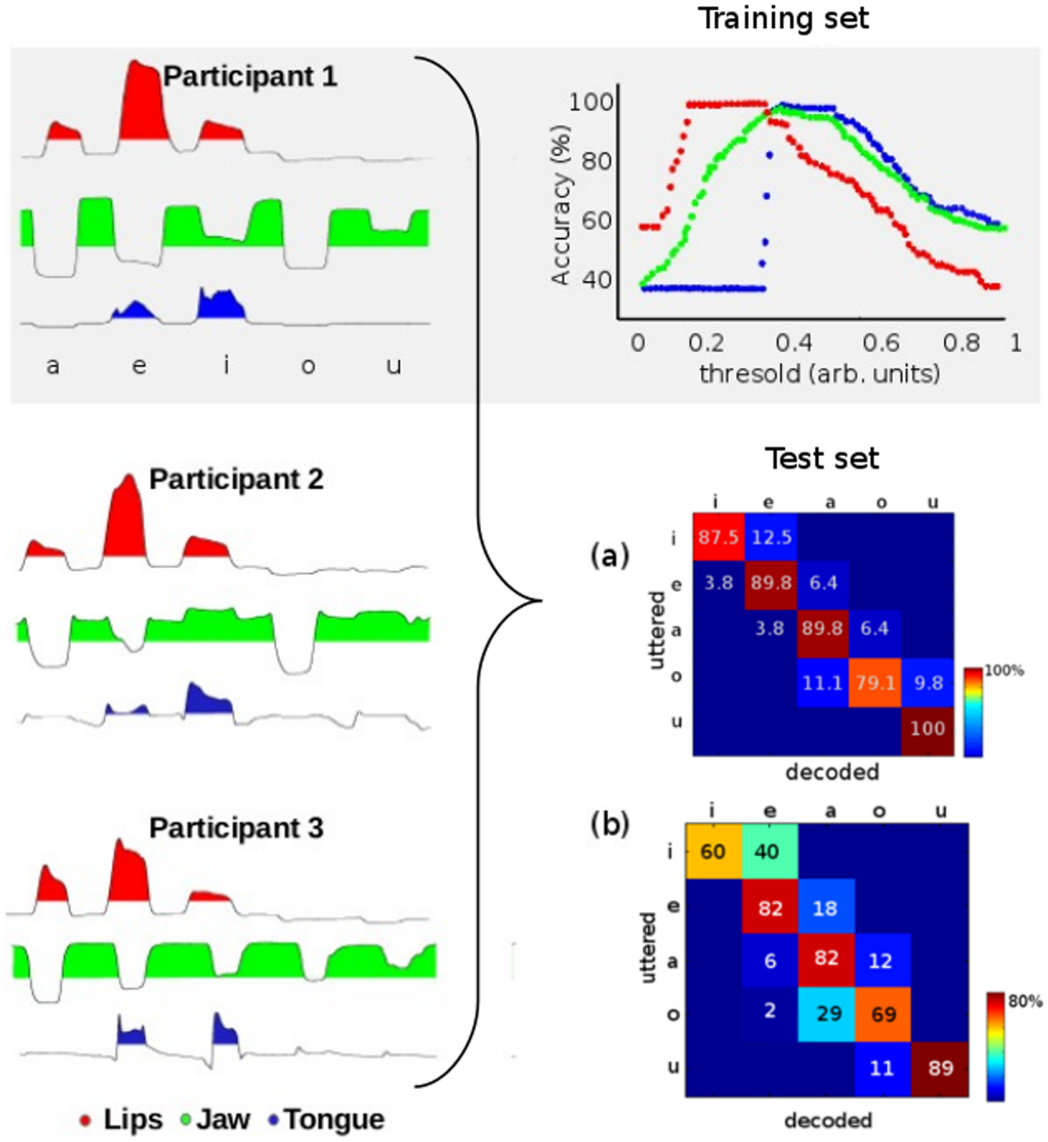
Decoding performances. Left: representative transducer signals during the utterance of the 5 Spanish vowels for the 3 participants. Up: decoding performance for each transducer across vowels using the training set for participant 1 (gray background). We show the hit rates for the lips (red), jaw (green) and tongue (blue) as a function of the threshold value. All the curves present high decoding accuracies over a range of threshold values. Lower right: confusion matrices across subjects for the test set. In (a), we set the thresholds at optimum values for each participant. In (b) we use a single set of thresholds for all the subjects.

In order to test the robustness of this description as a recognition system for Spanish vowels, we calculated the decoding performance for a dataset obtained from a population of different participants and recording sessions. In the left panels of Figure 2 we show examples of these signals.

Since our representation depends on the value of thresholds to assign discrete states to continuous vocal tract data, we first had to establish the optimum threshold values for each transducer before calculating the decoding performance. For this sake, we split our dataset into halves: we used one half as a *training set* that we used to optimize the threshold values, and the other half as a *test set* for calculating the decoding performance of our system (see Methods: recording sessions and training/test sets).

### Training set

We investigated the decoding performance of each transducer as a function of the threshold value. In Figure 2 (gray background), we show the decoding performance across vowels for participant 1. We note that the decoding accuracy of the 3 transducers, lips (red), jaw (green) and tongue (blue), present a similar behavior: the accuracy first increases until high decoding levels are reached and maintained over a range of threshold values, and then decreases to low accuracy values. This behavior was observed for the 3 participants, for which high decoding performances (>90%) were obtained at the following threshold ranges:

Participant 1: lips = (0.11, 0.40), jaw = (0.28, 0.55), tongue = (0.36, 0.62) (Figure 2).

Participant 2: lips = (0.05, 0.35), jaw = (0.4, 0.65), tongue = (0.41, 0.73),

Participant 3: lips = (0.21, 0.38), jaw = (0.2, 0.54), tongue = (0.38, 0.63).

These results indicate two different effects. On one hand, we have a common effect for every participant. High decoding performances are obtained over a range of threshold values, which simply means that slight variations of the transducer signals do not affect the vowel recognition or, in other words, that each vowel is compatible with a family of continuous transformations of the vocal tract.

On the other hand, the ranges of high accuracy are located at different threshold values for each participant. This inter-subject variability can be due to multiple causes: the different relative sizes of female and male vocal tracts, different speaking habits and also differences in the mounting of the transducer-magnet pairs. Any application of this representation as a decoding device must take into account these effects, and therefore we analyze this subject-dependent problem by calculating the decoding performance in two different situations.

### Test set

#### 1. Decoding performance using individual threshold values

For each participant, we set the threshold values at the center of their ranges and calculated the decoding performance across subjects on the *test set*. In Figure 2(a) we show the confusion matrix obtained in this case, showing high decoding performances for individual vowels and a small percentage of decoding errors that are limited to the band diagonal elements. These band diagonal elements correspond to the first neighbors of the correct vowel in the abstract motor coordinates (cube of Figure 1). In this way, the decoding errors correspond to vowels differing in just one digit from the correct one.

This shows the plausibility of a vowel recognition system based on binary motor gestures. An application of this method should then begin with a brief calibration period used to extract the thresholds from a given user, which are then used to classify new utterances into the different vowels.

#### 2. Decoding performance using common threshold values

We capitalize from the fact that the individual threshold ranges overlap for all the participants, and use a single set of thresholds at the intersection of individual ranges. The decoding performance for this case is shown in Figure 2b. Although the accuracy is lower than the one with individual thresholds, it is still well above chance (20%). In this case, a vowel recognition system using this discrete representation can be used without any calibration period.

So far, we presented a plausible strategy to reliably contract the motor space of continuous variables to a finite set of discrete states during the pronunciation of Spanish vowels. From the global perspective of the vocal program, this space of motor instructions can also be interpreted as an intermediate state between the two extremes of the vowel production, starting at the neural coding and ending at the vocal tract dynamics. We therefore examined how our representation can be used to link both extremes of the vocal program.

Recently, Tankus et al. revealed the neural codification for vowel production [4]. They found neurons in the Superior Temporal Gyrus (STG) responding broadly to all the pronounced vowels, from which they inferred a neural neighborhood structure for each vowel, which is analogous to the motor neighborhood shown in the confusion matrix of Figure 2a. More interestingly, the authors reported sharp-peaked tuned neurons in the rAC/MOF brain areas, exhibiting a strong increase in their spiking rates during the production of vowels. The total of neurons studied responded only to one or two vowels, while for the others, spiking remained at baseline. Based on these findings, we constructed a plausible neural codification matrix *V_N_*, where vowels are arranged in columns (from the left,/a/,/e/,/i/,/o/and/u/), and the 4 rows represent the neural populations from which the reported neurons were measured, either firing (1) or at baseline (0). 
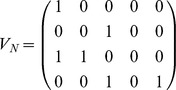



Downwards, rows correspond to the reported neural populations, firing exclusively during the production of/a/,/i/, (/a/,/e/), and (/i/,/u/). We are interested in connecting this binary neural matrix to our binary motor representation, in such a way that any column of *V_N_*, representing the neural code for a vowel, is transformed into the motor representation of that vowel. We proceeded by constructing the motor matrix *V*_M_, arranging in columns the vectors of the vowel-cube of Figure 1. Downwards, rows correspond to lips, jaw and tongue transducers:
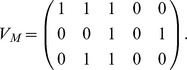



Conversion from neural to motor instructions is then accomplished using the simplest possible algebraic operation connecting two vector spaces: a linear transformation. In this case, 
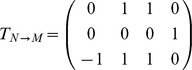
through the operation *T_N_*→*_M_ V_N_* = *V_M_*.

In this way, the neural activity driving the production of vowels is converted to the corresponding motor instructions in the abstract vowel space. On the other extreme of the vocal production stands the control of the vocal tract. In this case, we are interested in connecting the motor discrete space *V_M_* with the anatomical space of the different vocal tract configurations for each vowel.

Although the vocal tract is reconfigured in a continuous manner during speech, its shape is constrained by the different articulators and cannot be modified arbitrarily. A very elegant way to account for these anatomical constraints on the vocal tract movements can be found in the series of works [5]–[7]. The basic idea is to decompose the vocal tract shape into a few spatial functions. These functions are calculated from an empirical corpus of vocal tracts corresponding to different vowels, measured with magnetic resonance techniques. In this way, a vocal tract defined by a cross-sectional diameter *d* is approximated by the empirical orthogonal mode decomposition *d*(*x*) ≈ Ω(*x*) + *q*_1_ *φ*_1_(*x*) + *q*_2_ *φ*_2_(*x*). In this expression, *x* is the distance from the glottis to the lips, Ω(*x*) a neutral vocal tract function and {*φ*_1_(*x*), *φ*_2_(*x*)} are the first two spatial modes of the orthogonal decomposition.

The spatial modes *φ* do not correspond to specific vocal tract shapes, but rather to abstract space functions that can be linearly combined to generate any vowel shape through the abstract coefficients *q* in the low-dimensional space (*q*_1_, *q*_2_). This result suggests that it is possible to find other low-dimensional variables describing the vocal tract shape, as the experimental variables *V*_M_ described in this work. In order to connect both spaces, we calculated *v^i^*_A_ = (*q*_1_, *q*_2_)*^i^* for each Spanish vowel *i* (Table 1). We then found a simple way to connect the 3-dimensional motor space to the 2-dimensional anatomical space, through the affine map *T*_M→A_ *v^i^*_M_ + *a* = *v^i^*_A_, where *v^i^_M_* is the vowel *i* in the motor space (column *i* of *V_M_*). The map is:
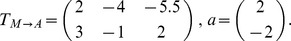



**Table 1:** Connecting the vowel formants to anatomical coefficients.

	<F_1_>	<F_2_>	(q_1_, q_2_)	
/a/	0.80	1.26	4	1
/e/	0.57	2.06	−1.5	3
/i/	0.28	2.50	−5.5	2
/o/	0.53	0.99	2	−2
/u/	0.31	0.77	−2	−3

We use the mapping described by Story et al. [6] to find the coefficients (*q*_1_, *q*_2_) corresponding to the first average formants *F*_1_ and *F*_2_ (kHz) of the Spanish vowels pronounced by our participants. This allowed the construction of a simple affine map connecting each Spanish vowel from the discrete motor space to their corresponding vocal tract configuration.

In this way, the motor and the anatomical spaces are connected by a simple linear map *T*_M→A_ followed by an abstract translation *a* between both spaces. This closes the loop needed to design a brain-machine interface for vowel production.

## Discussion

In this work we present a simple strategy for converting continuous anatomical changes in the upper vocal tract during the utterance of Spanish vowels to a discrete motor space of binary coordinates.

Our two most important findings are:

1. At the motor level, we showed that the mapping from continuous to discrete coordinates is robust for different speakers and recording sessions, which demonstrates the plausibility of constructing a vowel recognition system from a small set of anatomical gestures.

We also show that our representation is robust to inter-subject variations, at least for the group of participants analyzed here. Although Hall-effect transducers represent a good compromise between low cost and high temporal resolution, this technology responds nonlinearly to the relative distances of the articulators, complicating an exhaustive exploration of the sources of inter-subject variability, which would be useful to optimize the performance and the design of the detectors' configuration. A natural next step in this direction would be the incorporation of a different technology, as the mechanically invisible devices that have been recently developed allow acquisition of direct physiological data through multi-functional sensors [8].

Although much work is needed to generalize these results to other languages, we note that the dimension of the abstract motor space is 8, which is compatible with other attempts to characterize a basis for the vocalic space. Notably, the primary cardinal vowels are a subset of 8 vowels defined from a mixture of articulatory and auditory criteria [9], used by phoneticians to collapse all the possible vowel sounds into this well-defined reference set, and then describe each specific vowel as a particular perturbation of its closest cardinal. Given that the Spanish vowels map to different cardinal vowels, our discrete vowel space suggests a novel and simple alternative way to build an articulatory representation for 8 possible basic vowel states.

1. At the global level of vowel production, we showed that our discrete representation is a plausible link connecting the brain coding to the dynamics of the effectors. This is a qualitatively different alternative to the current models of human vocal production that capture peripheral dynamics in speech [10], [11], which require large dimensional measurements of the neural activity that are mapped into equally complex motor gestures.

Although the data used here from different studies and subjects may not be straightforwardly generalized, we show that neural, motor and anatomical data can be consistently connected with our representation through simple algebra, supporting a coherent view of neural control and production of vowels in terms of discrete motor states.

## Methods

### Participants

Three subjects (1 female and 2 males) within an age range of 35±6 years and no motor or vocal impairments participated in the recordings of anatomical and speech sound data. They were all native Spanish speakers, graduate students at the University of Buenos Aires, and signed a written consent. All the experiments described in this paper were approved by the ethics committee *Comité de Ética del Centro de Educación Médica e Investigaciones Clínicas* ‘*Norberto Quirno*’ (*CEMIC*) qualified by the Department of Health and Human Services (HHS, USA): IRb00001745 - IORG 0001315.

### Experimental setup

Removable, thin plastic dental casts (1 mm thick) were used by the participants during the experiments (dental casts of the superior and inferior dentures of each participant were supplied by the participants' dentists). Hall effect transducers (Ratiometric Linear Hall Effect Sensor ICs for High-Temperature Operation, A1323 Allegro) and small biocompatible Sm-Co magnets (1–3 g) were mounted in 3 different positions of the upper vocal tract for each participant (see figure 1). The transducer wires (Subminiature Lead Wire TDQ 44, Phoenix Wire Inc.) coated with a plastic tube (Silastic, Laboratory Tubing 0.76 mm×1.65 mm) were connected to a variable amplifier (2–30x), low-pass filtered (20 Hz) and recorded with a computer. Transducer wires and magnets were glued to the plastic replica using cyanoacrylate glue (Crazyglue, Archer, Fort Worth, TX) when needed. Denture adhesive (Fixodent Original Denture Adhesive Cream 2.4 Oz) was used to attach magnets to the tongue and medical paper tape (3 M Micropore Medical Tape) to fix the transducers to the lips.

The 3 magnet-transducer pairs were configured as sketched in figure 1:

- *Lips*. A cylindrical magnet (3.0 mm diameter and 1.5 mm height) was glued to the dental cast between the lower central incisors. The transducer was attached at the center of the lower lip.
- *Jaw*. A spherical magnet (5.0 mm diameter) and transducer were glued to the dental casts, in the space between the canine and the first premolar of the upper and lower teeth respectively.
- *Tongue*. A cylindrical magnet (5.0 mm diameter and 1.0 mm height) was attached at a distance of about 15 mm from the tip of the tongue, using a small amount of denture adhesive. The transducer was glued to the dental plastic replica, at the hard palate, approximately 10 mm right over the superior teeth (sagittal plane). Transducer wire was glued to the plastic replica and routed away to allow free mouth movements.

In this way, 2 out of 3 magnet-transducer pairs remained attached to the dental casts, which allowed an easy mounting and dismounting of the setup.

### Recording sessions and definition of the training/test sets

Four recording sessions were taken for each participant during a period of one month. Participants were asked to pronounce vowels separately from each other, starting from (and ending to) a comfortable closed mouth configuration. In each session, participants pronounced the vowels that appeared in random order a computer screen. The procedure was repeated until 20 sets of vowels were recorded at each session.

From the complete pool of 240 vowel sets (20 sets per session and participant) we randomly selected half for the training set and the other for the test set.

The Spanish vowels used in this work (/ä/,/e_T_/,/i/,/o_T_/and/u/) either coincide or differ slightly from the ones used in [4] (/α/,/ε/,/i/,/o/and/u/).

## Supporting Information

Dataset S1 **Compressed audio (wav) files of Spanish vowels for 3 participants.**
(ZIP)

Dataset S2 **Compressed lips transducer signals corresponding to S1 audio files.**
(ZIP)

Dataset S3 **Compressed jaw transducer signals corresponding to S1 audio files.**
(ZIP)

Dataset S4 **Compressed tongue transducer signals corresponding to S1 audio files.**
(ZIP)
